# Response to letter to the editor by Dr. Tomoyuki Kawada: “Carotid intima-media thickness and cardiovascular risk in patients with diabetes mellitus type 2 and chronic kidney disease”

**DOI:** 10.1080/0886022X.2020.1754854

**Published:** 2020-04-26

**Authors:** George S. Georgiadis, Christos Argyriou, Athanasios Roumeliotis

**Affiliations:** a Department of Vascular Surgery, “Democritus” University of Thrace, University General Hospital of Evros, Alexandroupolis, Greece; b Department of Nephrology, “Democritus” University of Thrace, University General Hospital of Evros, Alexandroupolis, Greece

To the Editor,

It is well known that vascular calcification is highly prevalent in patients with chronic kidney disease (CKD) and predicts a variety of cardiovascular (CV) disease events. Additionally, patients with diabetes mellitus type 2 (DM2) present a high prevalence of CV events due to accelerated vascular calcification, although the underlying pathogenic mechanisms are not completely understood. However, the combination of CKD and DM2 elevates the risk of both CV disease and death in this high-risk population [1]. Baseline intima-media-thickness of the carotid artery wall (cIMT) had been used as a marker of subclinical atherosclerosis and established as a surrogate marker for CV events, including death, in many population-based studies. However, longitudinal studies in patients suffering DM2 and CKD are scarce and occasionally with opposing findings.

We read with interest Dr. Kawada’s letter expressing important and insightful comments [2] about the effect of cIMT on CV morbidity and mortality in patients with CKD and DM2 identified in our study [3]. We also appreciate Dr. Kawada’s concerns about the precision of the Cox proportional hazard model regression analyses, specifically the use of 14 potential confounders (classic atherosclerotic risk factors) that did not satisfy the rule of 10 events per covariate [4]. In our view, some points need clarification.

Dr. Kawada mentioned for comparison in the letter [2], an excellent large observational study [5] in which cIMT and plaques, were measured at baseline and in regular follow-ups, in 478 participants with DM2 who were followed-up for a median of 10.8 years. Unfortunately, in that study, carotid imaging examinations for the evaluation of cIMT were performed by a single vascular sonographer specialist and hence, some systematic measurement error cannot be ruled out. Results of multivariate analyses for the risks associations between baseline early carotid atherosclerosis parameters and incident CV and renal events [new microalbuminuria development or new renal failure development (defined as doubling of serum creatinine or end-stage renal disease needing dialysis or death from renal failure)] and mortality outcomes during follow-up, revealed that most cIMT [(common carotid artery IMT (ccIMT) and internal carotid artery IMT (icaIMT)] parameters predicted total CV events occurrence, with excess risks varying from 15% to 18% for each 0.10 mm increase in cIMT, none carotid atherosclerosis parameters predicted all-cause or cardiovascular mortality, while only icaIMT (per 0.10 mm adjusted cIMT difference) and the increased plaque scores, predicted adverse renal outcomes (15% and 63% excess renal risk). Notably, only icaIMT >0.8 mm was associated with a 55% higher risk of developing a renal outcome (microvascular outcome) [5]. In our Cox regression models, both higher cIMT and lower eGFR represented independent prognostic factors for shorter life expectancy. Additionally, higher cIMT and albuminuria levels conferred prognostic value to a new CV episode. More specific, DM2-CKD patients with cIMT values >0.86 mm, had a greater risk of death (hazard ratio of 2.9) or experiencing a new CV event (hazard ratio of 2) during the 7-year follow-up period [3]. Indeed, we found higher risks for death and new CV events, however, we included patients with DM2 in different CKD stages (44.4% in stage 2, 38% in stage 3 and 4, mean eGFR = 47.6 mL/min/1.73m^2^) [3], in contrast to Cardoso et al. study in which only 29.3% of the whole cohort had diabetic nephropathy (mean eGFR in the all patients recruited = 82 mL/min/1.73m^2^) [5], suggesting different characteristics of the target subjects, which otherwise would have been welcomed in studies from different racial/ethnic groups. Notably, in another study from Asia, cIMT was also found a strong predictor of CV disease in Chinese pre-dialysis patients (∼88% with stage 3 and 4 of CKD which is less frailty than our corresponding subset of patients), indicating that increase in cIMT occurs early in the course of CKD [6]. Going further, Cardoso et al., recognized that the inclusion of middle-aged to elderly high cardiovascular risk DM2 individuals (mean age = 60 y in contrast to ours which was 68 y) is a study limitation [5] and so the results may not be generalizable to other diabetic populations. As Dr. Kawada correctly pointed out [2], the severity of diabetic nephropathy hinders precise prediction of CV events and all-cause mortality. Furthermore, the increased CV risk in patients with diabetic nephropathy cannot be solely explained by traditional risk factors. Besides biochemical (e.g.pro-inflammatory cytokines and endothelial dysfunction molecules), lifestyle risk factors, genetic factors also contribute to CV risk [3].

Regarding the methodology, we agree with Dr. Kawada [2] that the inclusion of >10 independent variables for the prediction requires a larger cohort and more extensive data with more endpoints for the analysis. However, in analysis of causal influences in observational data like ours, control of confounding may require adjustment for more covariates than the rule of 10 or more events per predictor variable allows. Of course, bigger samples and more events (minimum 140 would have been needed in our analyses) are almost always preferable to keep the validity of the statistical model. Therefore, many more events would have been needed for sub-analysis when stratifying the renal outcomes by cIMT, and further studies are warranted, as Dr. Kawada recommends [2]. Accordingly, enlarging the number of events with more patients and longer follow-up could permit for example separate examination of myocardial infarctions and strokes (both assigned as CV events in our study). However, <10 events per independent variable is also acceptable in some situations in logistic regression analysis, and there is no gold standard for simulation models to check the validity of the statistical outcome [7]. Vittinghoff and McCulloch reported that, situations commonly arise where confounding cannot be persuasively addressed without violating the rule of thumb [7], and we are aware that some other important predictors of CV event or patient survival (e.g. use of statins, smoking, unstable carotid plaques, calcification scores e.t.c.) could have been missed by our study (i.e. type II statistical error). Therefore, our results should be interpreted not with hostility and, in addition, compared with those from models from which weaker predictors have been excluded.

Finally, Dr. Kawada [2] cited another Asian article (surprisingly also cited in our article) [8], to emphasize that apart from baseline values of cIMT, cIMT progression in patients with DM2 without history of CV events, confers an up to 2.24 times higher risk for future CV events. Notably, the combination of baseline cIMT (≥1.1 mm) and cIMT progression (≥0.01114 mm/year) had a strong predictive power for CV events. eGFR correlated negatively with such events and patients developing study endpoints, had significantly lower mean eGFR. In that study [8] only DM2 patients were included and theoretically, the annual change of cIMT which reflects the progression of the atherosclerotic process should be a good marker of CV events also in DM2/CKD patients, a concept which fits our cohort. However, as the authors recognized, measurement of cIMT progression is more difficult than single measurement because random measurements errors at baseline and multiple follow-ups are aggregated [8]. Therefore, small quantity of cIMT progression might not be detected easily. Additionally, in contrast to our study, the authors evaluated only ccIMT including plaque [8], strengthening the fact that the use of different methods to measure cIMT or different parameters, yield different results, as those previously described in the articles cited by Dr. Kawada [2]. Apart from the above limitations, we agree that change of cIMT should be included for the risk assessment, which is otherwise still a controversial topic, but repeated cIMT measurements by the same ultrasonographer in regular clinical setting is difficult in some institutions. It is unclear, and possibly the thesis of a future scientific work, if higher annual changes of cIMT in patients with CKD and DM2, like our cohort, may allow a more accurate assessment of progression of atherosclerosis increasing the sensitivity of cIMT progression for the prediction of CV events and mortality.

Taken in aggregate, owing to its observational nature, our study cannot address all these open questions and we agree that further studies are needed to confirm our findings. We thank Dr. Kawada sharing his thoughts with us.

## References

[1] Roumeliotis S, Roumeliotis A, Panagoutsos S, et al. Matrix Gla protein T-138C polymorphism is associated with carotid intima media thickness and predicts mortality in patients with diabetic nephropathy. J Diabetes Complicat. 2017;31(10):1527–1532. 10.1016/j.jdiacomp.2017.06.012 28734846

[2] Kawada T. Carotid intima-media thickness and cardiovascular risk in patients with diabetes mellitus type 2 and chronic kidney disease. Ren Fail. 2020;42(1):314–314. 32216510 10.1080/0886022X.2020.1745237 PMC7170314

[3] Roumeliotis A, Roumeliotis S, Panagoutsos S, et al. Carotid intima-media thickness is an independent predictor of all-cause mortality and cardiovascular morbidity in patients with diabetes mellitus type 2 and chronic kidney disease. Ren Fail. 2019;41(1):131–138. 30909780 10.1080/0886022X.2019.1585372 PMC6442115

[4] Peduzzi P, Concato J, Feinstein AR, et al. Importance of events per independent variable in proportional hazards regression analysis. II. Accuracy and precision of regression estimates. J Clin Epidemiol. 1995;48(12):1503–1510. 8543964 10.1016/0895-4356(95)00048-8

[5] Cardoso CRL, Salles GC, Leite NC, et al. Prognostic impact of carotid intima-media thickness and carotid plaques on the development of micro- and macrovascular complications in individuals with type 2 diabetes: the Rio de Janeiro type 2 diabetes cohort study. Cardiovasc Diabetol. 2019;18(1):2. 10.1186/s12933-019-0809-1 PMC6327523 30630491

[6] Szeto CC, Chow KM, Woo KS, et al. Carotid intima media thickness predicts cardiovascular diseases in Chinese predialysis patients with chronic kidney disease. JASN. 2007;18(6):1966–1972. 17494886 10.1681/ASN.2006101184

[7] Vittinghoff E, McCulloch CE. Relaxing the rule of ten events per variable in logistic and Cox regression. Am J Epidemiol. 2007;165(6):710–718. 17182981 10.1093/aje/kwk052

[8] Concato J, Peduzzi P, Holford TR, et al. Importance of events per independent variable in proportional hazards analysis. I. Background, goals, and general strategy. J Clin Epidemiol. 1995;48(12):1495–1501. 8543963 10.1016/0895-4356(95)00510-2

